# Experimental Study and Model Simulation of the Effective Thermal Conductivity of Polymer/Carbonaceous Nanocomposites

**DOI:** 10.3390/nano16181162

**Published:** 2026-09-15

**Authors:** Panagiotis A. Klonos, Apostolos Kyritsis, Evagelia Kontou

**Affiliations:** 1 Physics Department, School of Applied Mathematical and Physical Sciences, National Technical University of Athens, 15780 Athens, Greece; pklonos@central.ntua.gr (P.A.K.); akyrits@central.ntua.gr (A.K.); 2 Mechanics Department, School of Applied Mathematical and Physical Sciences, National Technical University of Athens, 15780 Athens, Greece

**Keywords:** thermal diffusivity, polymer nanocomposites, model simulation

## Abstract

Improving the thermal conductivity of polymer/carbonaceous nanocomposites is still an interesting topic and is affected by a number of factors, such as polymer/nanofiller interaction, dispersion quality and agglomerate formation. In the present work, several polymer/nanocomposite types based on two different linear low-density polyethylenes (LLDPEs), as well as polylactic acid (PLA), reinforced with carbon nanotubes (CNTs) or a combination of CNTs/carbon nanofibers (CNFs) with graphene oxide (GO) at various loadings were investigated. The thermal diffusivity results for monofiller and hybrid nanocomposites with varying nanofiller loadings were analyzed. Additionally, existing models were employed to simulate the thermal conductivity using experimental data of both monofiller and hybrid nanocomposites. The model parameter values were utilized to explore the mechanism of the thermal conductivity increase, the role of CNTs and the synergistic effects of hybrid nanocomposites.

## 1. Introduction

Over the last few years, there has been an increased interest in polymers with high thermal conductivity in the field of electronic packaging in microelectromechanical systems. The generation of a large amount of heat requires an efficient high-heat-dissipation rate [1]. At the same time, the satisfactory performance of devices demands good electric isolation; therefore, the substitution of metallic materials by high-thermal-conductivity polymers is an interesting task. The design of thermally conductive polymer composites provides new opportunities for substituting metallic parts in power electronics, heat exchangers, and electric motors/generators, owing to these polymers’ benefits, namely, a lightweight nature, processability and oxidation resistance.

During the last few decades, several types of carbon-based nanomaterials, including carbon nanotubes (CNTs), carbon nanofibers (CNFs) and graphene nanoparticles (GO), have been utilized as additives in bulk polymeric matrices to adapt their mechanical, thermal, and electrical properties for a number of specialized technological applications [2,3,4,5]. This is a consequence of their outstanding structures and special properties, such as low density, high aspect ratio [6], high modulus and tensile strength [7] and remarkable electrical [8] and thermal conductivities [9].

Regarding the enhanced thermal conductivity of polymer/carbonaceous nanocomposites, a number of works have been published so far [10,11,12,13,14,15,16,17].

The selection of the most suitable nanofiller with high thermal conductivity to be dispersed in the polymeric matrix is an issue of great interest. As reported in reference [18], CNTs appeared to be the most promising conductive nanofillers, but the thermal conductivities of polymer/CNT nanocomposites did not meet expectations. This effect can be attributed to the high interfacial thermal resistance between CNTs and the surrounding polymer, which restricts the transfer of hot phonons between the highly conductive CNTs and the poorly conductive polymer. It has been proposed that the actual thermal percolation for thermally conductive nanofillers is theoretically expected to occur at very high loadings (e.g., above 30% [18,19]), which is highly unrealistic due to processing and economic costs. Also, in the case of semicrystalline polymer nanocomposites, it has been proposed that the role of nanofillers (including CNTs and GO) is indirect, acting via the imposed effects on the semicrystalline morphology and polymer crystal interconnectivity (ordered structures) [13,20,21,22].

As reported in reference [23], CNTs and graphene nanoplatelets (GNPs) are attracting considerable interest as a novel form of thermal fillers in polymer composites applied in thermal interface materials. This is due to their one-dimensional and two-dimensional structures, their high aspect ratio and their high thermal conductivity, which may result in a remarkable thermal conductivity enhancement of the nanocomposite.

It was found that the low thermal conductivity of polymers at room temperature, approximately 0.1–1 W/m·K, can be improved by the dispersion of carbonaceous nanoparticles [17,24]. The thermal conductivity of CNTs ranges between 2000 and 3000 W/m·K, while that of GO is between 4840 and 5300 W/m·K [25,26]. As a consequence, polymer/carbonaceous nanocomposites may be appropriate for heat scattering or cooling of microelectronic, battery and solar cell devices [27,28,29]. Significant improvement in thermal conductivity was detected upon the incorporation of CNTs or GNPs into a polymeric matrix [30]. The effective thermal conductivity of the nanocomposite is influenced by a number of parameters, namely, the size and geometry of the nanofiller, the dispersion quality, the nanofiller volume fraction and matrix–nanofiller adhesion quality.

Within this context, it is reported in reference [31] that the thermal conductivity of an epoxy could be increased by 125% with the addition of 1 wt% CNTs. Also, in reference [32], the thermal conductivity of 1-octadecanol increased by two times with the addition of graphene at a volume fraction of 4%.

The thermal transport of polymer nanocomposites has been investigated by a number of methods, namely, molecular dynamics [33,34,35], analytical [36] and micromechanical modeling [37,38], sometimes while also considering the aggregation effect [39,40].

In recent years, advanced polymer nanocomposites based on hybrid CNT/GO nanofillers have attracted increased interest. The combination of the two nanofiller types was found to lead to a synergistic effect, causing impressive changes in mechanical properties and thermal and electrical conductivities [3,4,36,41,42,43].

The binary mixture of GNPs and single-walled CNTs, at a 3:1 weight ratio, resulted in enhanced thermal conductivity compared to reinforcement with an individual nanofiller. Therefore, improved thermal conductivity can be obtained at a low nanofiller loading, associated with decreased viscosity and improved processability [18,44,45].

In reference [5], the mechanical and thermal properties of a thermoplastic natural rubber were improved by the incorporation of hybrid multi-walled CNTs (MWCNTs) and SiC nanoparticles as reinforcements and conductive fillers. This was accomplished by optimizing the amounts of the two different nanofillers, which resulted in a synergy between them.

Notwithstanding the large number of publications on such hybrid nanocomposites, a complete theoretical analysis of their thermal conductivity response remains insufficient due to the complex mechanisms involved in the synergistic effects of the two nanofillers [23]. A theoretical study of the thermal conductivity of hybrid nanocomposites based on CNTs/GNPs and incorporating the percolation effect is presented in reference [23]. Reasonable agreement between theoretical and experimental results was reported.

In the present work, a systematic experimental investigation of the thermal diffusivity of a variety of carbonaceous polymer nanocomposites has been performed. Polymer nanocomposites based on two types of polyethylene matrices reinforced with CNTs at various loadings, as well as hybrid nanocomposites based on polyethylene or polylactic acid (PLA) reinforced with a combination of graphene oxide (GO) with either CNTs or carbon nanofibers (CNFs) at an equal ratio and various total nanofiller loadings, were examined. There are not many relevant publications on this type of nanocomposite; therefore, the present research constitutes a new contribution to the field since it provides experimental results on thermal diffusivity/conductivity for polymer nanocomposites with different polymer matrix types and different combinations of carbonaceous nanofillers.

Furthermore, by performing experiments on the specific heat capacity and the material density, the thermal conductivity of the nanocomposites could be determined. Model simulation of thermal conductivity using existing theoretical equations developed for nanocomposites was conducted. Depending on the experimental thermal conductivity results, linear and nonlinear models, as well as models introduced for hybrid nanocomposites, were employed to describe/predict the effective thermal conductivity. Moreover, to incorporate the effect of the crystallinity increment due to the presence of the nanofillers, thermal conductivity variation with the nanofiller loading was assumed for the polymeric matrix. The experimental study offers new information about the factors influencing the thermal conductivity of nanocomposites, while the model simulation based on the experimental results and the subsequent discussion provide new evidence on the mechanisms of thermal conductivity enhancement.

## 2. Experimental Section

### 2.1. Materials

A variety of polymer/carbonaceous nanocomposites were employed for the experimental investigation of thermal diffusivity/thermal conductivity. The first series is based on linear low-density polyethylene (LLDPE) as a matrix material, reinforced with CNTs at various loadings. LLDPE was produced either by a metallocene catalyst, designated as mLLDPE, or by a Ziegler–Natta catalyst, referred to as zLLDPE. The mLLDPE, kindly supplied by Flexopack SA, Athens, Greece, has a density equal to 0.902 g/cm^3^, and the melt flow index (MFI) was 1.0 when measured at 190 °C at a load of 2.16 kg, according to ASTMD1238-65T [46]. Multi-walled carbon nanotubes (MWCNTs), provided by Aldrich (St. Louis, MO, USA), had, on average, an outer diameter of 10 ± 1 nm, an inner diameter of 4.5 ± 0.5 nm, and a length of 3–6 μm. Preparation details are reported in reference [47]. The zLLDPE matrix, sold under the commercial name SABIC LLDPE 118WJ, had a melt flow rate (MFR) of 1.0 at 190 °C at a load of 2.16 kg, according to ISO 1133 [48], while its density is 0.918 g/cm^3^. Multi-walled CNTs (MWCNTs) were synthesized by a chemical vapor deposition process and were supplied by Tinesnano Chengdu Organic Chemicals Co (Chengdu, Sichuan Province, China). The average diameter of CNTs was 10–20 nm, and their length was about 10 μm [49]. The designations and the nanofiller loadings of all nanocomposites examined are shown in Table 1.

The second series is based on an mLLDPE matrix reinforced with CNTs, CNFs and GO at one specific weight fraction, as well as with a combination of GO/CNTs and GO/CNFs at an equal ratio and various loadings [4]. The materials’ designations are shown in Table 2. The employed MWCNTs were the same as those in the first series, while graphene oxide (GO) was provided by United Nanotech Innovations PVT. LTD (Karnataka, India). and was functionalized with OH groups. As provided by the manufacturer, the number of layers was 3–6 on average, the average thickness in the Z dimension was 2–3 nm, and the average length in the X and Y dimensions was approximately 5 μm. Carbon nanofibers (CNFs), provided by Aldrich, had a length ranging between 20 and 200 μm, while the average diameter was 100 nm.

The third series was prepared with polylactic acid (PLA) as a matrix, reinforced with a combination of either GO/CNTs or GO/CNFs at various total loadings and at an equal ratio [3]. The PLA/nanocomposites’ designations are presented in Table 3. The polylactic acid utilized is sold under the commercial name Ingeo™ Biopolymer 2003D, produced by Nature Works LLC, Minnetonka, MN, USA, and was kindly supplied by the Greek Company M. Procos S.A., Oinofyta, Boeotia, Greece.

The selected grade 2003D has a density of 1.24 g/cm^3^ and an MFR index equal to 6 g/10 min, measured at 210 °C at a load of 2.16 kg, according to ASTMD1238-65T. The preparation procedure for the hybrid nanocomposites is presented in detail in reference [3].

### 2.2. Experiments

Differential scanning calorimetry (DSC) experiments on the polymer nanocomposites under investigation have been performed in our previous works [3,4,38,41]. The crystallinity degree of the neat matrices and the related nanocomposites are presented in Table 1, Table 2 and Table 3. The corresponding crystallinity enhancement due to the presence of nanofillers is listed as well.

Temperature-modulated calorimetry (TMDSC) was employed to accurately evaluate the heat capacity, *C_p_*, at room temperature. *C_p_* was actually taken as the reversing part of the total heat capacity. The measurements were conducted by employing a properly calibrated TA Q200 calorimeter (TA Instruments, New Castle, DE, USA) in a pure nitrogen atmosphere, using samples with a mass of ~12–15 mg, which were sealed in Tzero pans as they were heated from −5 to 35 °C at 2 °C/min, with a modulation period of 60 s and a 1 K amplitude.

The thermal diffusivity, α, was measured at 20 and 25 °C (room temperature-like) employing the light flash analysis (LFA) method in a nitrogen atmosphere, employing a NETZSCH LFA 467 HyperFlash apparatus (NETZSCH, Selb, in Bavaria, Germany). The samples were in the form of thin cylindrical pellets of 0.5 inch in diameter and 1.5–2 mm in thickness, spray-coated with graphite. Based on the LFA detector signal, α was estimated by employing the half-time method of Parker [50] via Equation (1):

(1) $$\alpha =1.38\cdot L^{2}/(\pi \cdot t_{1/2})$$

where *L* is the sample thickness, and *t*_1/2_ is the required time to reach the half-maximum temperature (signal) rise on the rear sample surface. A necessary parameter for performing the LFA measurement and, in the following, for estimating the thermal conductivity is the mass density. This was determined by dividing the measured sample mass by the corresponding volume. The repeatability of the measurement was checked by taking measurements on 2–3 samples per composition and by taking 3 continuous measurements (light pulse shots) for each sample. Based on the recorded data, the standard deviation of thermal diffusivity was estimated at δ*α*~0.002 mm^2^/s.

## 3. Results

The experimental results for the thermal diffusivity of the mLLDPE/CNT and zLLDPE/CNT nanocomposites under investigation are presented in Table 1. In addition, the percentage crystallinity of each material type is reported.

**Figure 1:**
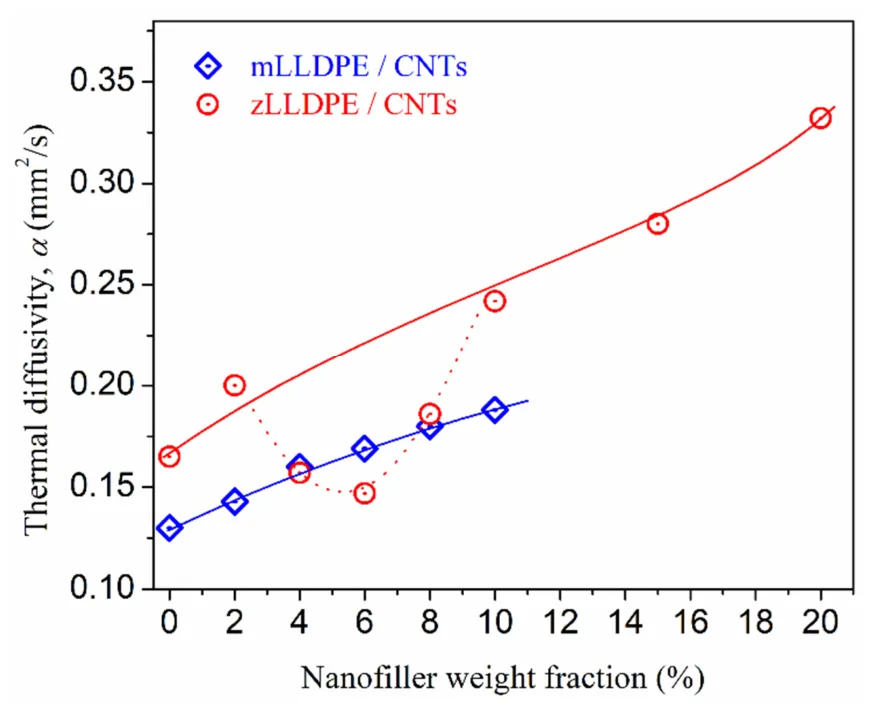
Thermal diffusivity results at room temperature for the mLLDPE/CNT and zLLDPE/CNT nanocomposites with varying nanofiller weight fractions. The added lines crossing the experimental points are used as guides for the eye.

Figure 1 comparatively illustrates the experimental thermal diffusivity results of the mLLDPE/CNT and zLLDPE/CNT nanocomposites with varying nanofiller weight fractions obtained at room temperature.

The CNT nanocomposites obtain higher values of thermal diffusivity than the mLLDPE matrix, which monotonically increases with increasing CNT weight fraction. As shown in Table 1, the percentage crystallinity of the nanocomposites exhibits an increasing trend with the CNT weight fraction. This result can be attributed to the large aspect ratio of the CNTs, which facilitates the formation of a heat-conductive network within the matrix. As mentioned in reference [5], the conductivity of the nanocomposites arises mostly from the heat transport of the highly conductive nanofillers. On the other hand, the increased crystallinity degree of the nanocomposites also plays an important role, since the low thermal conductivity of polymers is partly due to the low crystallinity levels that usually appear [1]. Regarding neat polymers, as reported in reference [1], one reason for their low thermal conductivity is the low crystallinity of their internal physical structure. For example, polyethylene with an 81% crystallinity degree has a 70% higher thermal diffusivity than polyethylene with a 43% crystallinity degree.

Concerning the second series of zLLDPE/CNT nanocomposites, as shown in Table 1 and Figure 1, generally, a similar trend is observed at high nanofiller loadings, with the exception of 4 and 6% CNTs, where lower thermal diffusivity values than those of the zLLDPE matrix are obtained. This anomalous response is attributed to the high interfacial thermal resistance between the matrix and nanofiller, associated with a number of factors, like agglomerate formation and the curvature and waviness of CNTs. A thermal expansion mismatch and poor chemical adhesion of the polymer to the particle surface may lead to inefficient transport of phonons through the interface. This is the so-called interfacial thermal resistance (Kapitza’s resistance of an interphase boundary) [18]. Comparing the two groups, it can be seen that the zLLDPE/CNT nanocomposites demonstrate higher thermal diffusivity values at the same nanofiller loadings, while the crystallinity degree is also higher. This behavior is in accord with the reinforcing effect of crystallinity on the thermal diffusivity trend. The polyethylene matrix type also seems to play a decisive role, given that the presence of CNTs promotes the formation of crystalline regions, especially in the zLLDPE matrix.

The second series of nanocomposites under investigation is based on monofillers, namely, mLLDPE reinforced with GO, CNTs, or CNFs at a constant loading equal to 1.31 wt%, as well as on a combination of GO/CNT and GO/CNF/hybrid nanocomposites at various total nanofiller loadings.

**Figure 2:**
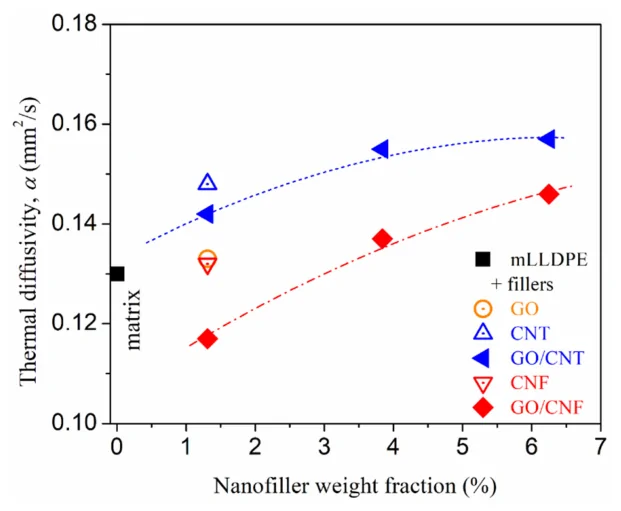
Thermal diffusivity results of mLLDPE/monofillers and mLLDPE/hybrid nanocomposites at room temperature with varying nanofiller weight fractions. Lines crossing the experimental points are provided to guide the eye.

The designations of the various types of nanocomposites and the experimental results for crystallinity degree and thermal diffusivity are presented in Table 2, while the thermal diffusivity results with varying nanofiller loadings are illustrated in Figure 2.

From Table 2 and Figure 2, it can be observed that the monofillers based on CNTs demonstrate higher thermal diffusivity values than the other monofiller types.

Comparing monofillers and hybrids at the same 1.31% nanofiller loading, it is observed that mLLDPE/1.31% CNT exhibits the highest value, while the other two monofillers demonstrate values between GO/CNT and GO/CNF hybrids. Furthermore, mLLDPE/GO/CNF 1.31% exhibits a lower thermal diffusivity than the matrix, similarly to zLLDPE/CNTs 4% and 6%, while thereafter it increases with increasing nanofiller loading.

The hybrid nanocomposites based on GO/CNTs demonstrate an incremental thermal diffusivity trend with increasing total nanofiller loading, which is higher than that exhibited by the GO/CNF hybrids. This effect can be attributed to the fact that CNTs, having high aspect ratios, may act as heat-conductive channels between GO particles [5]. As reported in reference [23], thermal conductivity enhancement can be obtained due to a percolation effect of graphene nanoplatelets triggered by the presence of CNTs, which does not occur with isolated CNTs and graphene oxide.

As shown in Table 2, the percentage crystallinity of all nanocomposites is higher than that of the neat matrix, with the nanocomposites based on GO/CNFs exhibiting the highest crystallinity values.

As far as crystallinity is concerned, it has also been reported in references [20,51] that the higher the crystallinity, the higher the thermal conductivity for various polyethylene types.

From the above results, it can be deduced that the thermal diffusivity of the polymers/nanocomposites arises from two sources: the thermal diffusivity of the carbonaceous nanofillers and crystalline regions into the polymer bulk. In some cases, asynergy between these two factors may lead to an enhancement of the thermal diffusivity, whereas in some other cases, due to the nanofillers’ special geometry (CNFs versus CNTs), no synergetic effect is possible [13,19,21,22].

It is noted that the thermal conductivity values of the polymer nanocomposites examined are limited by the high interfacial thermal resistance (Kapitza resistance) between the nanotubes and the matrix, preventing the full realization of the intrinsic high conductivity. CNTs’ functionalization can reduce the Kapitza resistance, while poor dispersion and agglomerate formation lead to a reduction in thermal diffusivity [52].

When nanofillers are poorly dispersed, they form isolated clusters that create heat flow barriers (phonon scattering), rather than effective conductive paths. It seems that the Kapitza resistance is enhanced in the case of GO/CNF hybrid nanocomposites.

As reported in ref. [4], mLLDPE/GO/CNT nanocomposites exhibit lower mechanical enhancement than mLLDPE/GO/CNF hybrids at the same nanofiller loadings. This can be attributed to the fact that blending GO and CNFs results in improved dispersion and better adhesion between the mLLDPE matrix and nanofillers. However, from the thermal diffusivity results, it can be inferred that the interfacial thermal resistance between the matrix and CNFs, in the presence of GO platelets, is higher than the resistance between the matrix and CNTs in the GO/CNT hybrids.

Furthermore, comparing the behavior of the mLLDPE/hybrids with the results of the mLLDPE/CNT monofiller nanocomposites (Figure 2), it can be observed that the presence of the GO platelets promotes the formation of isolated clusters, which subsequently hinder the formation of thermally conductive paths.

The experimental results for crystallinity and thermal diffusivity of the third series of nanocomposites, namely, PLA/carbonaceous hybrid nanocomposites, are presented in Table 3.

**Figure 3:**
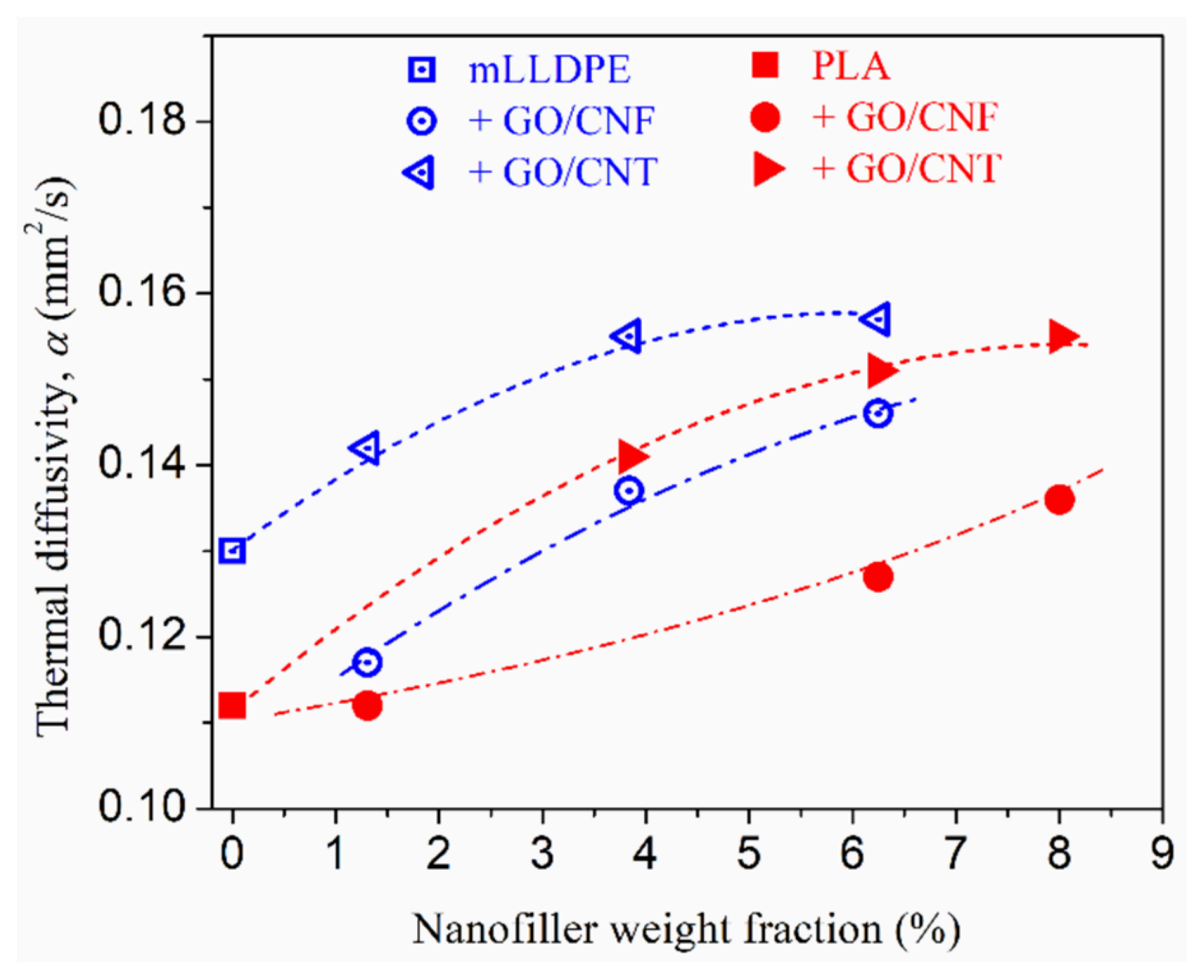
Comparative plots of the experimental thermal diffusivity results at room temperature with varying nanofiller weight fractions of PLA/hybrid and mLLDPE/hybrid nanocomposites.

In Figure 3, the thermal diffusivity of PLA/hybrids is comparatively depicted with the corresponding experimental results for the mLLDPE/hybrid nanocomposites.

In Table 3 and Figure 3, it is observed that the PLA/GO/CNT hybrids have higher thermal diffusivity than the corresponding GO/CNF hybrid nanocomposites.

The percentage thermal diffusivity increment is 38.4% for PLA/GO/CNT/8% and 21.2% for PLA/GO/CNF/8%. These values are quite comparable to the thermal conductivity increment of PLA/GNPs reported in ref. [14]. Regarding the crystallinity content of the PLA/hybrids, a detailed analysis of the differential scanning calorimetry (DSC) results is presented in [3]. The overall crystallization kinetics result from an interplay between the nucleating effect and the growth disturbance effect of the filler particles.

It can be seen in Table 3 that the percentage crystallinity of the PLA/hybrid nanocomposites increases in the presence of nanofillers, particularly for PLA/GO/CNTs, which also exhibit the highest thermal diffusivity enhancement compared with GO/CNFs at similar nanofiller loadings. This behavior reveals that there is a synergy between crystallinity and CNTs involved in the improvement in thermal conductivity.

In other words, it is confirmed here that the thermal diffusivity of the polymer/nanocomposites arises from two sources: the thermal diffusivity of the carbonaceous nanofillers and crystalline regions into the polymer bulk.

In addition, the GO/CNT hybrids exhibit higher thermal diffusivity than the GO/CNF formulations for all polymeric matrix types investigated.

## 4. Theoretical Study of the Effective Thermal Conductivity of Monofiller and Hybrid Nanocomposites

The thermal conductivity *K* can be calculated by the following equation:

(2) $$K=\alpha \cdot \rho \cdot C_{p}$$

where α is the thermal diffusivity (mm^2^/s), *ρ* is the density (g/cm^3^), and *C_p_* is the specific heat capacity (J/m·K). The experimental results for thermal conductivity of zLLDPE/CNT nanocomposites and the mLLDPE and PLA/hybrid nanocomposites are presented in Table 4. It can be seen from Table 4 that the thermal conductivity values of the zLLDPE/CNT nanocomposites are higher than that of the neat matrix, with the exception of the 6% loading, which exhibits a value that is about 2% lower. This is the result of incorporating the material’s density and specific heat values into the calculation of thermal conductivity.

### 4.1. Model Simulation of the Thermal Conductivity of the zLLDPE/CNT Nanocomposites

In this section, the thermal conductivity of the zLLDPE/CNT nanocomposites is evaluated by Equation (2) on the basis of the experimental data on thermal diffusivity, heat capacity and density and is analyzed using a model developed in previous work [17]. The model is based on two converging approaches. The first approach [53] takes into consideration the effect of the carbon nanofiller type and its geometrical features, while the second one [54] introduces a nonlinear relation between the thermal conductivity and the nanoparticle volume fraction, resulting from Monte Carlo simulation and percolation theory. The obtained equation for the effective thermal conductivity, *K_e_*, of a polymer/CNT nanocomposite is given by

(3) $$K_{e}=K_{m}\left[1+\frac{{\left(f-\frac{0.6}{p}\right)}^{n}}{3}\left[\frac{1}{H+{\left(\frac{K_{z}}{K_{m}}-1\right)}^{-1}}\right]\right]$$

where it is assumed that the critical volume fraction *f_c_* is equal to 0.6/*p*, with *p* being the nanofiller’s aspect ratio. *K_m_* and *K_z_* are the thermal conductivities of the matrix and the CNTs respectively, *n* is an exponent to be fitted, and *f* is the nanofiller’s volume fraction. *H*(*p*) is an expression of the nanofiller’s aspect ratio, calculated as follows:

(4) $$H(p)=\frac{1}{p^{2}-1}\left[\frac{p}{\sqrt{p^{2}-1}}\ln\left(p+\sqrt{p^{2}-1}\right)-1\right]$$

The effective conductivity of CNTs, *K_z_*, is given by Equation (5),

(5) $$K_{z}=\frac{K_{f}}{1+2\frac{R_{k}K_{f}}{L}}$$

where *R_k_* is the interfacial thermal resistance at the filler–filler and filler–matrix interfaces, *K_f_* is the intrinsic thermal conductivity of CNTs, and *L* is the length of CNTs.

The experimental data on thermal conductivity of zLLDPE/CNTs, as shown in Table 4, were employed to validate this model. At the beginning of this study, the effects of the main model parameters, namely, the CNTs’ aspect ratio, the CNTs’ length, the parameter *n* and the resistance *R_k_*, were investigated. It is noted that the CNTs’ nominal dimensions are 10 μm in length and 10–20 nm in diameter.

**Figure 4:**
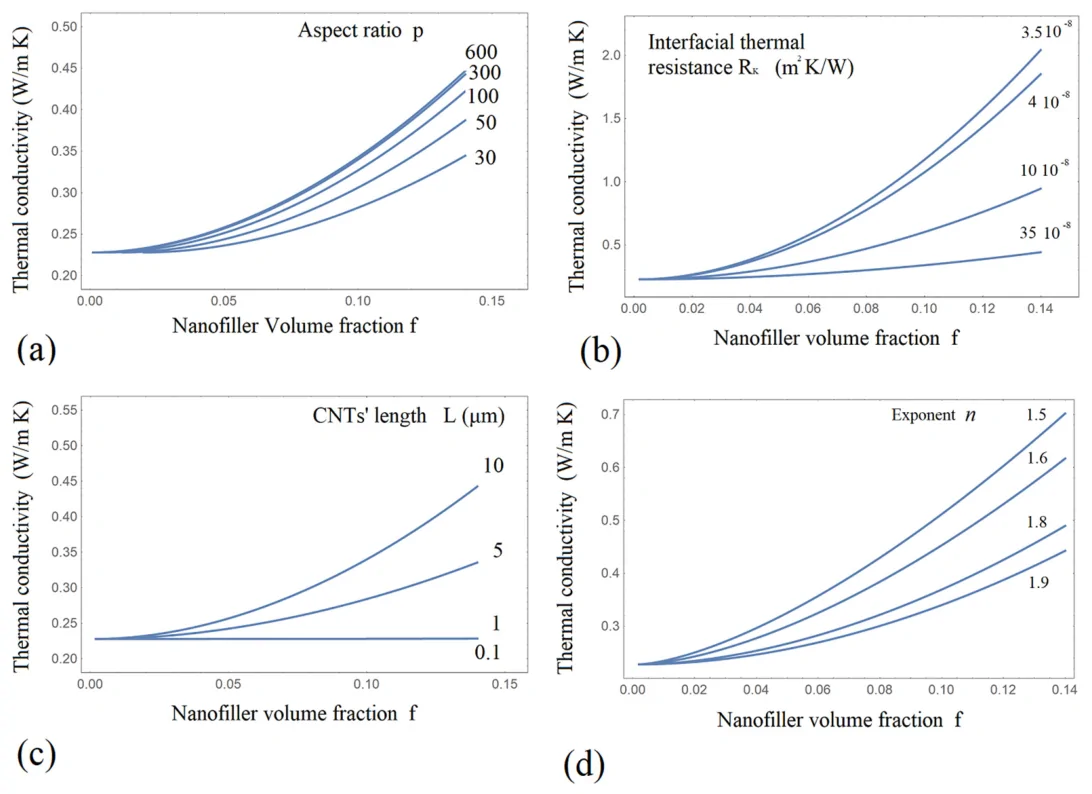
Model (Equation (3)) parameter values’ effects on the thermal conductivity of the polymer/CNT nanocomposite, namely, (**a**) aspect ratio, (**b**) interfacial thermal resistance, (**c**) CNT length and (**d**) exponent *n*.

In Figure 4, the variation in thermal conductivity with varying nanofiller volume fractions at several values of these parameters is depicted.

Based on the above parametric analysis, the model simulation of the experimental data on thermal conductivity was conducted using the following parameter values: *K_f_* = 2000 (W/m·K), *K_m_* = 0.228 (W/m·K), *R_k_* = 3.5 × 10^−7^ (m^2^ K/W), *p* = 300, *L* = 10 μm and *n* = 1.9.

**Figure 5:**
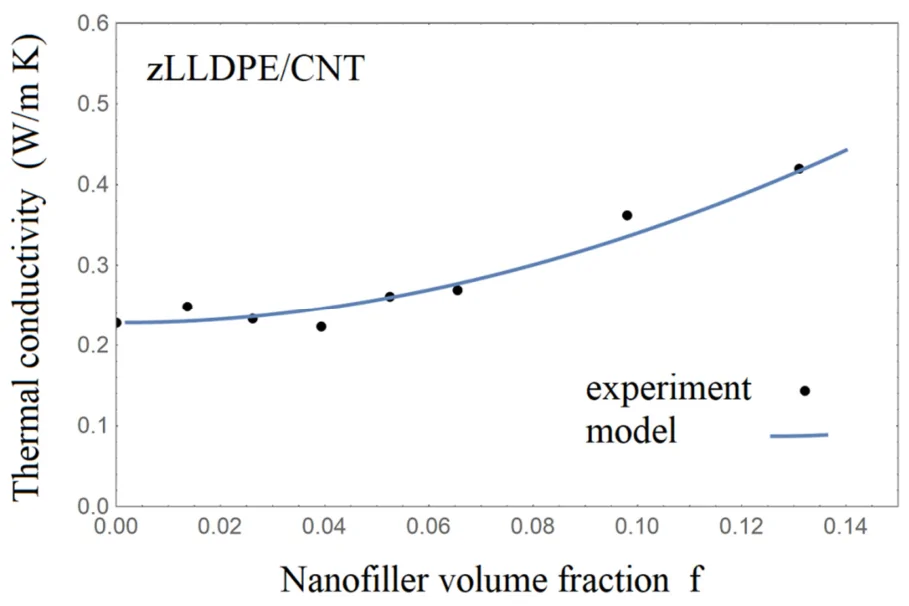
Thermal conductivity values of the zLLDPE/CNT nanocomposites with varying CNT volume fractions. Points: experimental data; lines: model (Equation (3)) simulation.

The experimental values of thermal conductivity for the zLLDPE/nanocomposites are comparatively plotted with the model simulation results in Figure 5.

From this plot, quite satisfactory agreement between experimental data and model simulation results has been achieved. It should also be mentioned that the obtained lowering of the thermal diffusivity of the nanocomposites (Figure 1) compared to that of the neat matrix is less intense in the thermal conductivity results. This anomaly can be associated with a number of factors that govern the experimental thermal conductivity results and may have contradictory effects, as will be discussed in the following paragraphs.

In ref. [49], where the zLLDPE/CNTs were extensively experimentally investigated, it was found by SEM that at a low CNT loading, a sparse and non-uniform distribution of CNTs is obtained. With increasing CNT loading, agglomerates with increasing size could be observed. For example, for zLLDPE/CNT 8%, the agglomerate size was on the order of 145 nm, whereas the size could reach 700 nm, on average, at a higher CNT loading. In conclusion, a rather satisfactory CNT distribution—found to be better at high CNT contents—coexisting with smaller and larger agglomerates can be obtained. The SEM results are therefore considered to be in accord with the anomalous trends of thermal diffusivity (Table 3) and thermal conductivity (Table 4) at the low CNT loadings.

### 4.2. Model Simulation of the Thermal Conductivity of Hybrid Nanocomposites

In the following, the experimental thermal conductivity results for the mLLDPE/hybrid and PLA/hybrid nanocomposites, as presented in Table 4, will be analyzed by an appropriate model.

In reference [23], a theoretical study of the effective thermal conductivity of hybrid CNT/GNP composites, within the framework of the effective medium, was carried out by incorporating the percolation effect. Within the framework of this model, good agreement between theory and experimental data was confirmed. This model is capable of describing the synergistic response of thermal conductivity enhancement obtained with CNT/GNP hybrid nanocomposites. Following this analysis, an approximated form of the effective thermal conductivity of a hybrid nanocomposite *K*^∗^ is given by the following equation:

(6) $$K^{*}=K_{m}\left[\frac{1+\frac{f_{c}}{3}\left(\frac{K_{c}^{eff}}{K_{m}}\right)+2\frac{f_{g}}{3}\left(\frac{K_{g}^{eff}}{K_{m}}\right)}{1-\frac{\left(2f_{c}+f_{g}\right)}{3}}\right]$$

where *K_m_* is the effective thermal conductivity of the matrix, *f_c_* and *f_g_* are the volume fractions of CNTs and GNPs respectively, and $K_{c}^{eff}$ and $K_{g}^{eff}$ are the effective thermal conductivities of CNTs and GNPs and given by the following equations:

(7) $$K_{c}^{eff}=\frac{K_{c}}{2\frac{R_{k}K_{c}}{L_{c}}+1}$$

(8) $$K_{g}^{eff}=\frac{K_{g}}{2\frac{R_{k}K_{g}}{L_{g}}+1}$$

where *K_c_* and *K_g_* are the thermal conductivities of the CNTs and GNPs respectively, *L_c_* and *L_g_* are the length and width of CNTs and GNPs, and *R_k_* is the filler–matrix interfacial thermal resistance, which is a parameter of great importance.

From the thermal conductivity results of the nanocomposites under investigation, the model was proved to be capable of satisfactorily predicting/describing the thermal conductivity of the PLA/GO/CNF and mLLDPE/hybrid nanocomposites. The model parameter values are shown in Table 5.

**Figure 6:**
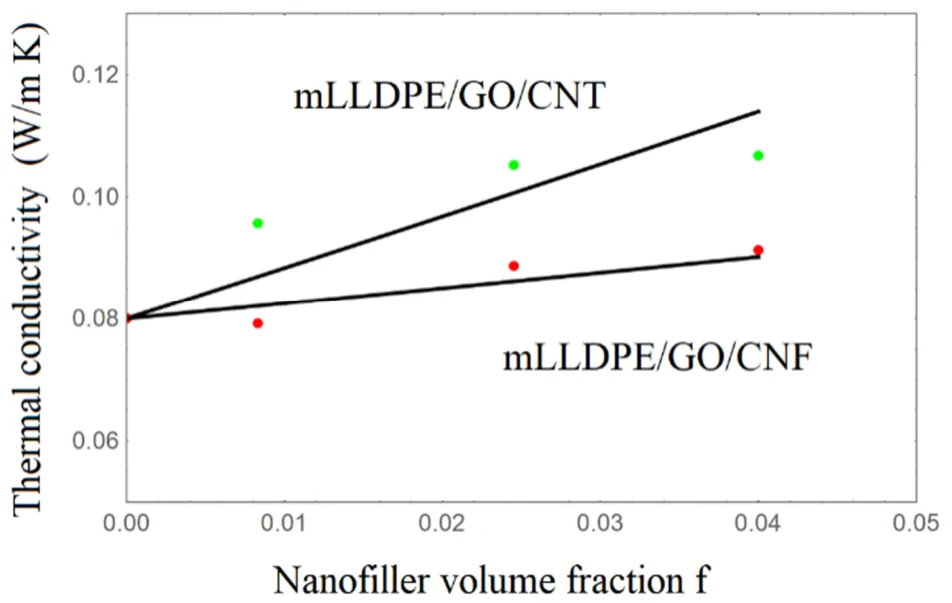
Thermal conductivity values of the mLLDPE/hybrid nanocomposites with varying total nanofiller volume fractions. Points: experimental data; lines: model (Equation (6)) simulation.

**Figure 7:**
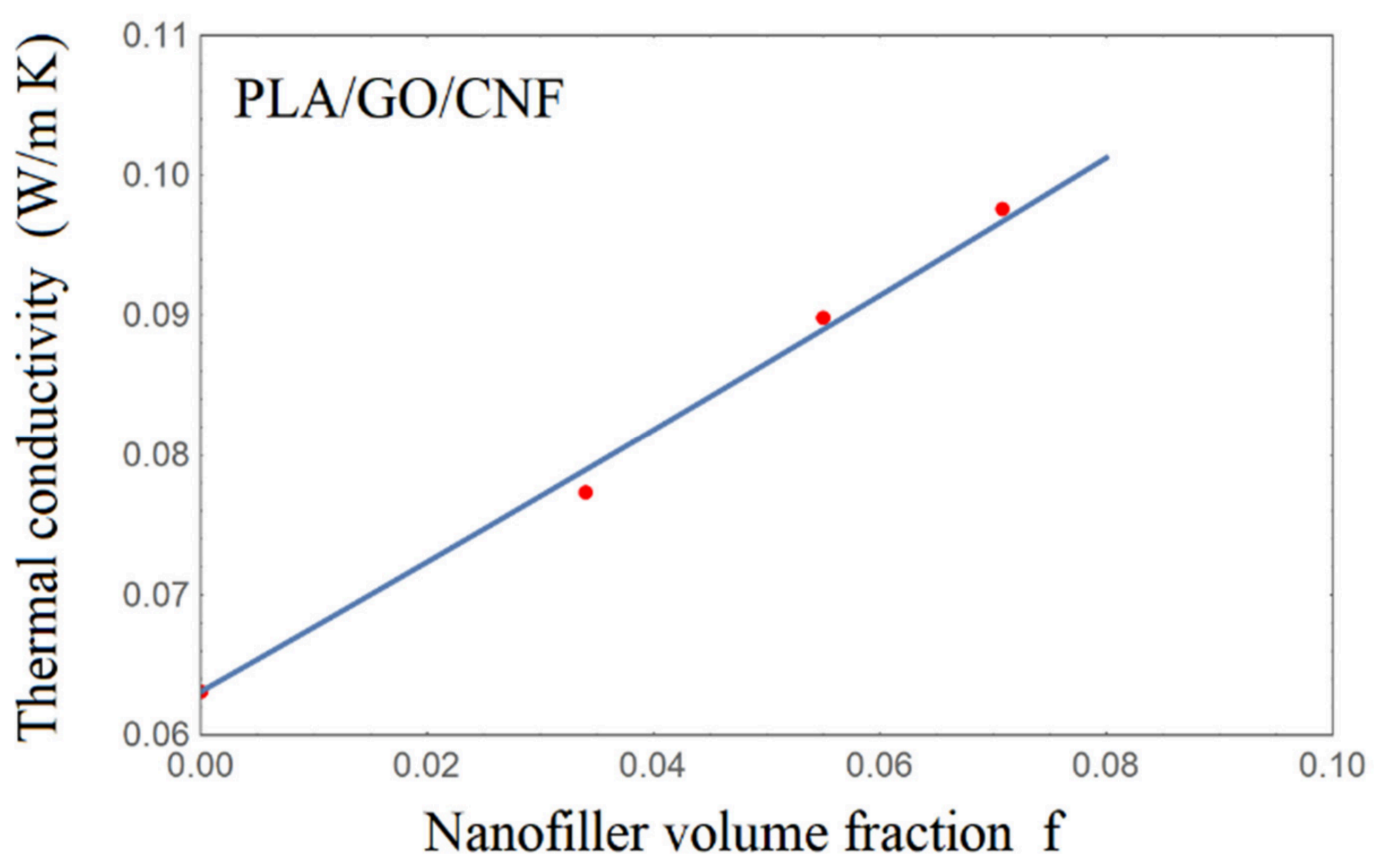
Thermal conductivity values of the PLA/hybrid nanocomposites with varying total nanofiller volume fractions. Points: experimental data; lines: model (Equation (6)) simulation.

The experimental thermal conductivity data are compared with model simulation results in Figure 6 and Figure 7.

As shown in Figure 6, a discrepancy is observed for mLLDPE/GO/CNF/1.31 wt%, which mainly arises from the low experimental value of thermal diffusivity, and the final value obtained is also affected by the density and heat capacity values. The lower thermal diffusivity compared to that of the pure matrix is related to the increased interfacial thermal resistance (Kapitza resistance) between the CNFs and the matrix, preventing the complete realization of the intrinsic high conductivity. The agglomerates also play an important role in this behavior. As shown in Figure S1 in the Supplementary Material (SM), for mLLDPE/GO/CNF/1.31%, GO flakes coexist with CNFs, which are sparsely dispersed. This image is in accord with the obtained discrepancy in thermal conductivity. As far as the higher nanofiller loadings are concerned, in Figure S2, for mLLDPE/GO/CNF/3.84%, a good CNF dispersion is observed with the GO flakes scattered in the matrix. Similarly, in Figure S3, for mLLDPE/GO/CNT/8%, large GO flakes partially attached to the polymer and a good CNF dispersion can be seen. As far as the mLLDPE/GO/CNT hybrid at 1.31% loading is concerned, in Figure S4, bundles of partially exfoliated GO flakes are seen, with CNTs being randomly distributed and seldom seen. With increasing nanofiller loading, well-dispersed CNTs are observed with GO flakes between them, coexisting with CNT bundles, as shown in Figures S5 and S6.

Regarding the model parameters listed in Table 5, it is noted that the employed values of the nanofillers’ thermal conductivity are taken from the literature [23].

The thermal conductivities *K_c_* and *K_g_* of the CNTs and GO respectively are both taken as equal to 2000 (W/m∙K), following ref. [23]. It is mentioned here that, regarding the thermal conductivity values of the nanofillers, it is generally accepted that these values exhibit significant variations, particularly at lower levels, depending on several factors, such as the dispersion quality and the nanofiller’s orientation. For example, the measured thermal conductivity values of graphite in the basal plane range from 900 to 2000 W/m∙K, and those normal to the basal plane are two or three orders lower [54]. The selected value of 2000 W/m∙K for both nanofiller types was considered to be the value falling within the range of values found in the literature.

Furthermore, it is important to mention that the same value for the parameter *R_k_* was assumed for CNT/matrix and GO/matrix, given that GO and CNTs are related materials [23,55]. Additionally, the dimension of GO was taken as equal to 5 μm, within the range of its nominal dimensions, and then the parameters *R_k_* and *L_c_* could be back-calculated. As shown in Table 5, the dimensions of CNTs and CNFs employed are in the range of the nominal values; therefore, these values are not included in the fitting procedure. The thermal conductivity of mLLDPE/GO/CNTs is higher than that of mLLDPE/GO/CNFs, indicating the presence of efficient bridging between GO and CNTs.

Given the complex morphology of the materials under investigation, the experimental thermal conductivity results are dominated by several factors that either promote or retard thermal conductivity. Therefore, the experimental results represent a balance between the effects of these factors.

As far as the interfacial characteristics of the GO/CNT and GO/CNF systems are concerned, the high aspect ratio of CNFs has a retarding effect on thermal conductivity (more pronounced interfacial thermal resistance); therefore, GO/CNTs exhibit a better conductivity response. As an additional observation, the effect of agglomerates may be considered. The two series of materials were studied in a previous study of ours [40], where a semi-empirical model was used for the calculations. It was found that the PLA/GO/CNTs at high loadings had higher agglomerate volume fractions, rich in nanofillers. This effect resulted in better thermomechanical performance.

It is noted, therefore, that the estimated *R_k_* values, mainly for the mLLDPE/GO/CNF and PLA/GO/CNF hybrids, are much higher than the ones reported for different types of nanocomposites. The low thermal conductivity and, consequently, the high interfacial thermal resistance calculated are associated with the curvature and waviness of the CNTs/CNFs, which has been confirmed, as representatively depicted in Figure S7. To the best of our knowledge, there are no published experimental data for similar hybrid nanocomposites that would allow for a comparison. Application of the model revealed that to achieve *R_k_* values on the order of 10^−7^, it is necessary to use *L_c_* and *L_g_* values that are one or two orders of magnitude lower. This can be seen in a relative plot in Figure S8 in the SM. Furthermore, additional model calculations were performed to determine the specific roles of model parameters. A separate study was performed to analyze the effect of the special interfacial resistance value of the CNTs and GO, namely, the *R_kc_* and *R_kg_* quantities. The results are illustrated in Figures S9 and S10 in the SM, and it seems that the resulting thermal conductivity is more affected by small changes in *R_kg_*. It is noted that these plots were created with *L_c_* and *L_g_* equal to 0.5 μm (much lower than the nominal dimensions), and in spite of varying values of either *R_kc_* or *R_kg_*, the estimated values of *R_k_* of the nanocomposites did not reach values as low as those recorded in the literature. As already mentioned, in our analysis we chose to keep the *R_kc_* and *R_kg_* values equal, which is also the method followed in the literature. What is valuable at this stage is the comparative study of the thermal conductivity values of the different types of hybrid nanocomposites studied in the present work and the interfacial thermal resistance value required to achieve the optimal fitting.

### 4.3. Nonlinear Model for the Thermal Conductivity of the PLA/GO/CNT Hybrids

**Figure 8:**
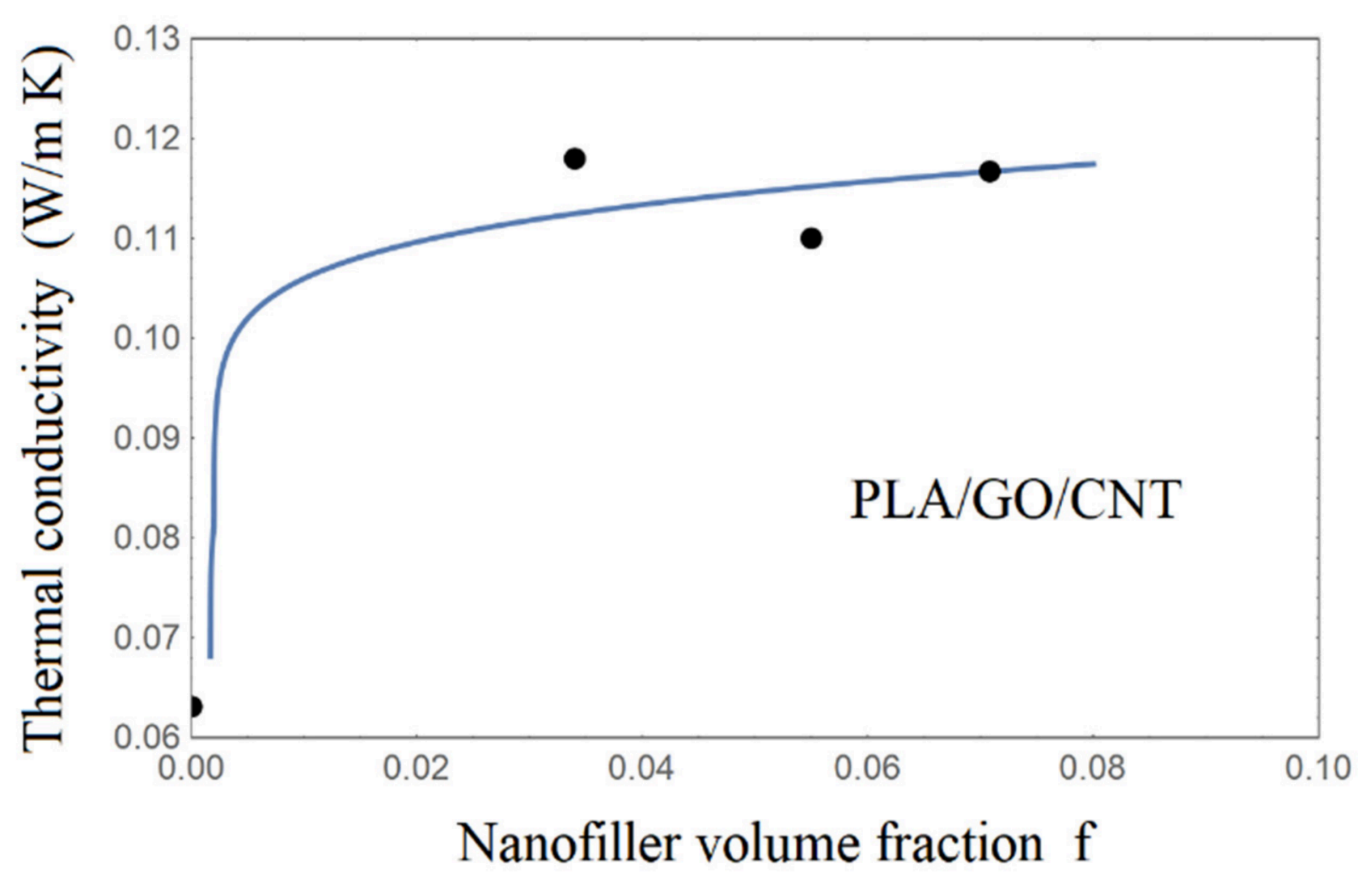
Thermal conductivity values of the PLA/GO/CNT nanocomposites with varying total nanofiller volume fractions. Points: experimental data; lines: model (Equation (9)) simulation.

The PLA/GO/CNT thermal conductivity, as depicted in Figure 8, exhibits nonlinear behavior, with an abrupt enhancement with varying nanofiller contents.

The abrupt change in the thermal conductivity slope with varying nanofiller weight fractions is usually considered an indication of the percolation threshold [56]. On the other hand, there has been a debate about whether percolation behavior appears in the thermal conductivity of carbonaceous nanocomposites [57]. Unlike the sharp electric percolation, a true distinct percolation for thermal conductivity in GNP/CNT nanocomposites is weak or does not exist. However, a gradual transition or pseudo-threshold typically appears between 0.5 vol% and 4 vol%. The percolation threshold depends on several factors, namely, the nanofiller’s aspect ratio (the higher the aspect ratio, the lower the percolation threshold) and the dispersion state. The uniform dispersion leads to network formation at higher loadings, while aggregates can cause localized early percolation. The synergy between nanofillers is a crucial factor for percolation.

It has been reported [58] that thermal percolation, defined as the rapid enhancement of *K* above a certain nanofiller content, can be caused by overlapping graphene nanoplatelets within the polymeric matrix. This effect is due to the conversion of the primary system governed by interfacial thermal resistance to a system dominated by contact thermal resistance, which is a result of phonon scattering at the inter-filler contacts. It has also been confirmed [59] that thermal conductivity can be significantly enhanced in GNP/CNT hybrid nanocomposites compared to the corresponding binary nanocomposites. It is also mentioned that the similarity between the nanofillers’ aspect ratios seems to be a main factor for exhibiting synergistic effects. The lower the aspect ratio difference, the higher the synergy obtained, which is associated with a reduced percolation threshold. In the same work, numerical results obtained by the Monte Carlo method revealed varying percolation threshold values for various CNT:GNP ratios, with an average of 0.008, while this value was 0.012 or 0.0195 for binary nanocomposites.

The thermal conductivity behavior in Figure 8 was described by the nonlinear version of the model in Equation (6). This version was introduced in ref. [23] to describe the nonlinearity and the higher values of thermal conductivity in a system of epoxy/CNT/GND nanocomposite. The nonlinear form of the effective thermal conductivity is given by the following equation:

(9) $$K_{\mathrm{nl}}^{*}\;={\;K}_{m}\left[\frac{1+\frac{f_{c}}{3}\left(\frac{K_{c}^{eff}}{K_{m}}\right)+2\frac{{\left(f_{g}\;-\;0.001\right)}^{\beta }}{3}\left(\frac{K_{g}^{eff}}{K_{m}}\right)}{1-\frac{\left(2f_{c}+f_{g}\right)}{3}}\right]$$

Assuming that the thermal conductivity of a graphene oxide nanoparticle is proportional to (*f* − *f_p_*)*^β^*, where *f_p_* is the percolation threshold, a low value of *f_p_* is adopted here. The nonlinear trend of the experimental results in Figure 8 leads to the conclusion that the percolation effect starts at very low nanofiller loadings; therefore, *f_p_* was taken as equal to 0.001. A similar procedure was followed in ref. [23].

The exponent *β* is a critical property of thermal conductivity; it represents structural information and depends on the intrinsic conductivity of the nanofillers and on the contacts between them [23], and it is treated here as a fitting parameter. The nonlinear model parameters are presented in Table 6, and a comparison between experimental data and model simulation results is illustrated in Figure 8. As shown in Figure 8, the trend of thermal conductivity with increasing nanofiller loading could be acquired satisfactorily. These results are in accord with the above-mentioned corresponding findings in the literature, and, therefore, a synergetic effect indication between CNTs and GO can be considered. It is noted that, as shown in Table 6, the CNT/GO dimensions that are included in the calculations have similarities.

Within the context of this nonlinear model [23], it has been suggested that a 3D percolating network can be formed by bridging interactions between GNPs and CNTs, thereby facilitating the heat flow. This contributes to a synergistic effect, where the presence of CNTs activates the percolation effect.

A similar synergistic effect for the PLA/GO/CNT hybrid nanocomposites was found in a previous work of ours [3] by broadband dielectric spectroscopy (BDS). Synergy between GO and CNTs was validated, resulting in the formation of conductive paths into the matrix. This was attributed to an improved nanofiller dispersion and a functional linkage between GO and CNTs. As a complementary and auxiliary observation in this analysis, the results of electron microscopy can be considered. Regarding the PLA/GO/CNΤ hybrids [3], a similar picture is seen at all nanofiller loadings, with the CNTs being well dispersed in all cases, coexisting with the exfoliated GO flakes.

These hybrids also demonstrated the highest mechanical enhancement compared to the PLA/GO/CNF nanocomposites. Therefore, although the focus is on the effects of different physical properties, it is worth noting the obtained synergy between GO and CNTs, reflected in both BDS results and thermal conductivity.

Referring to the hybrid nanocomposites studied in the present study, PLA/GO/CNT is the specific type that exhibits the highest thermal conductivity compared to the other hybrids under investigation. It is not possible to directly compare the thermal conductivities of the nanocomposites studied in this work with those of high-conductivity hybrids in the literature based on ceramic and carbonaceous materials. As an example of such a comparison, one could mention the composites in ref. [60], where ceramics and carbon-based fillers were incorporated into the polymeric bulk to obtain composite materials with competitive thermal conductivity. Hybrid fillers of CNFs and alumina nanospheres were dispersed in a polymeric blend of PLA and an ethylene–acrylic ester–glycidyl methacrylate terpolymer. The polymer composites with 15 wt% CNFs and 130 wt% ANSs exhibited thermal conductivity of 0.97 W/m∙K.

It is noted that in the present study, no further investigation was conducted regarding chemical interactions between the polymer matrix and the nano-inclusions.

Key interaction mechanisms between a polymeric matrix and CNTs or GO nanoplatelets occur through non-covalent or covalent bonding, having a decisive role in interfacial stress transfer, thermal transfer and stability and, consequently, in the nanocomposite’s entire thermomechanical and rheological response [61,62].

### 4.4. The Effect of the Crystallinity Increment on the Thermal Conductivity Model Simulation

As described in the previous paragraphs, an effort was made to simulate the thermal conductivity of the polymer nanocomposites under investigation by employing models developed for either monofiller or hybrid nanocomposites, having a linear or nonlinear form.

In all cases the thermal conductivity value *K_m_* of the polymeric matrix was considered to be constant. Given that crystallinity is one of the main sources of the thermal conductivity enhancement, as already mentioned in the previous text, in this section a modification of the employed models is made by incorporating the crystallinity increment while increasing the nanofiller volume fraction.

**Figure 9:**
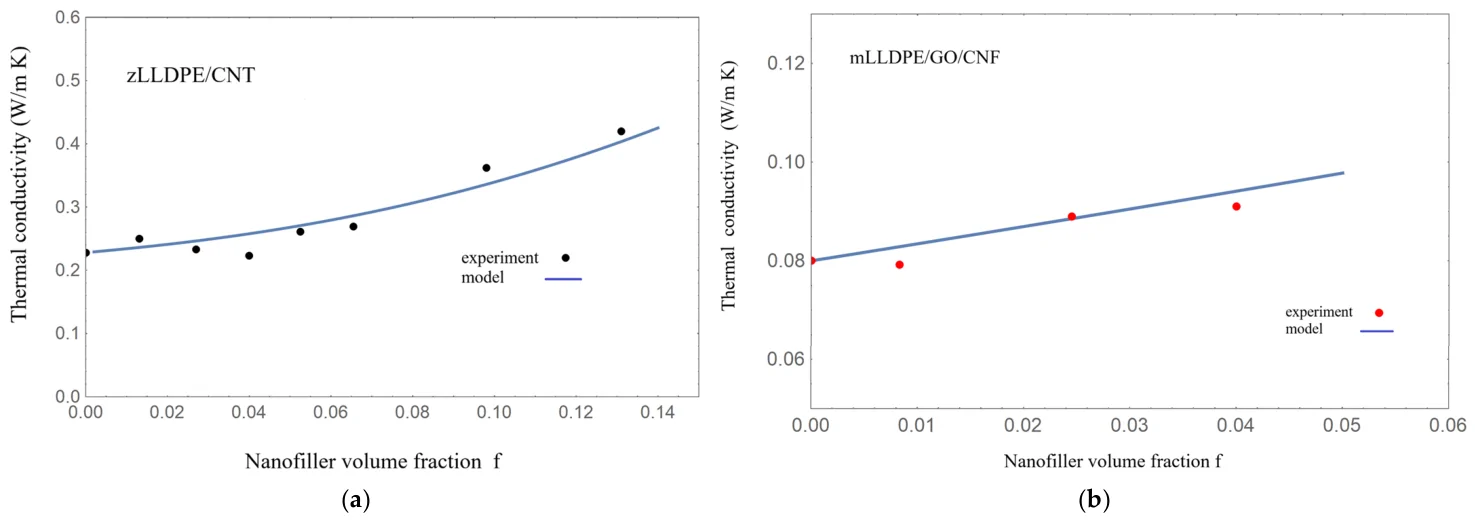
(**a**) Thermal conductivity values of the zLLDPE/CNT nanocomposites with varying total nanofiller volume fractions. Points: experimental data; lines: model simulation by modified Equation (3). (**b**) Thermal conductivity values of the mLLDPE/GO/CNF nanocomposites with varying total nanofiller volume fraction. Points: experimental data; lines: model simulation based on modified Equation (6).

In all cases, a simple approximation was made, namely, the linear dependence of the polymer’s thermal conductivity on the nanofiller volume fraction, *f*, in the form *K_m_*+ *k* · *f*, where *K_m_* is the neat polymer’s thermal conductivity, and *k* is a factor that needs to be estimated. Therefore, in the model equations, this expression provides the value for the polymer’s thermal conductivity. Regarding Equation (3), employed for the zLLDPE/CNT nanocomposites, the new calculations are shown in Figure 9a. The corresponding model parameters are as follows: *K_f_* = 2000 (W/m·K), *k* = 0.6 (W/m·K), *R_k_* = 3.0 × 10^−7^ (m^2^ K/W), *p* = 300, *L* = 10 μm and *n* = 2.3. Equation (6) was employed to simulate the experimental thermal conductivity results of the PLA/GO/CNF, mLLDPE/GO/CNF and mLLDPE/GO/CNT hybrids. According to Table 2 and Table 3, the percentage crystallinity changes with increasing nanofiller volume fraction are very small for PLA/GO/CNF and mLLDPE/GO/CNT. Therefore, we focused on the mLLDPE/GO/CNF nanocomposites. Following the same procedure as in Equation (3), the linear dependence of the polymer’s thermal conductivity with increasing volume fraction due to the crystallinity increment was assumed. The model parameter values are as follows: *K_f_* = 2000 (W/m·K), *K_g_* = 2000 (W/m·K), *k* = 0.08 (W/m·K), *R_k_* = 7.0 × 10^−5^ (m^2^ K/W), *L*_c_ = 100 (μm), *L*_g_ = 5 (μm). The model simulation results based on the modified Equation (6) and the experimental results are comparatively shown in Figure 9b.

**Figure 10:**
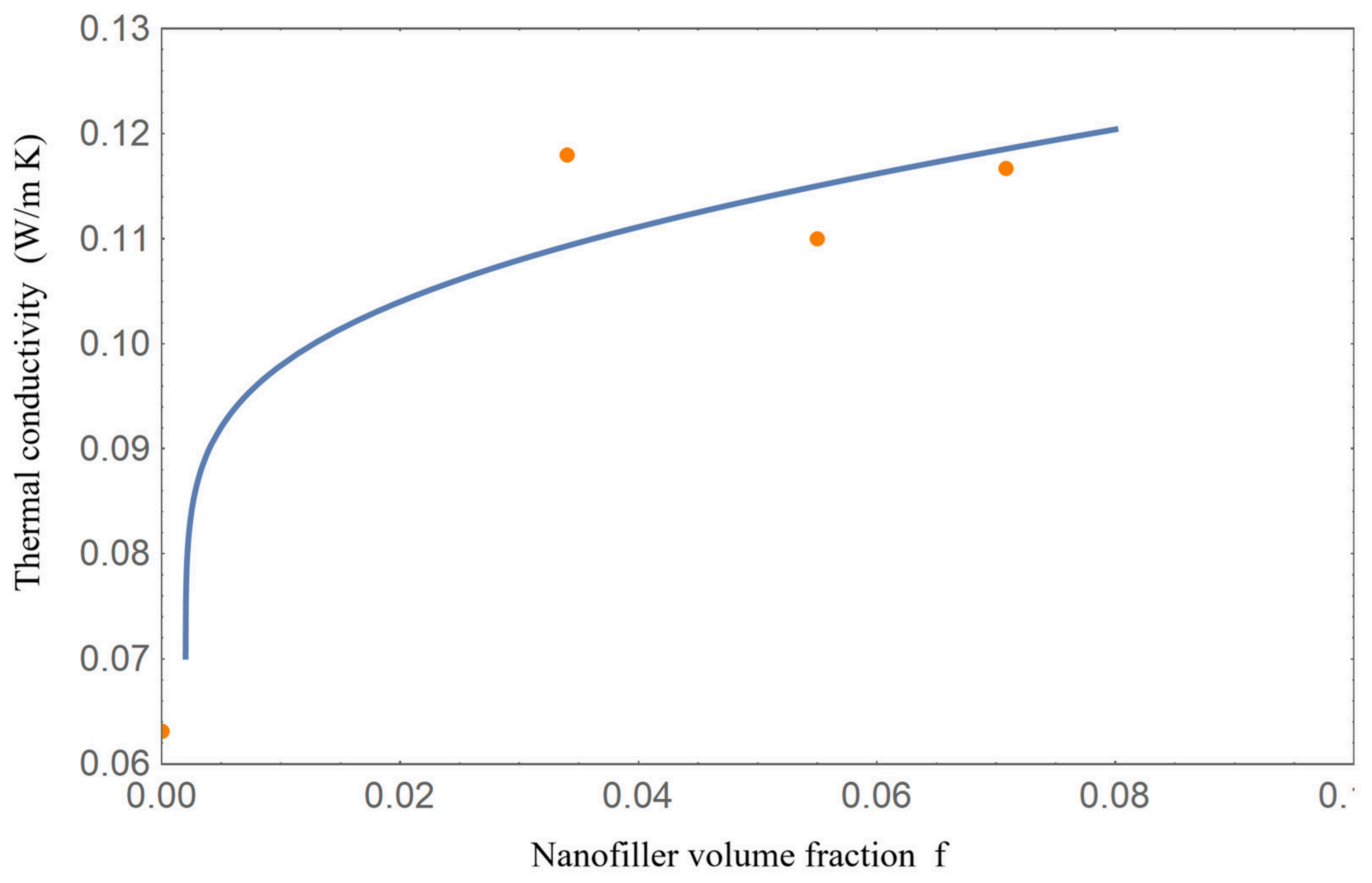
Thermal conductivity values of the PLA/GO/CNT nanocomposites with varying total nanofiller volume fraction. Points: experimental data; lines: model simulation based on modified Equation (9).

Equation (9) is actually the nonlinear form of Equation (6), and it is associated with the percolation threshold concept. It was employed to describe the PLA/GO/CNT series, which indeed exhibits a high crystallinity increment (Table 3), but it does not monotonically vary with increasing nanofiller loading. Assuming again the linear dependence of the polymer’s thermal conductivity on the nanofiller volume fraction, we incorporated the crystallinity variation into the thermal conductivity calculation. The model simulation results obtained with the modified Equation (9) and the experimental data are depicted in Figure 10.

The model parameter values are as follows: *K_c_* = 2000 (W/m·K), *K_g_* = 2000 (W/m·K), *k* = 0.02 (W/m·K), *R_k_* = 18 × 10^−6^ (m^2^ K/W), *β* = 0.18, *L*_c_ = 5 (μm), *L*_g_ = 5 (μm). It is noted that in all cases, quite satisfactory agreement between experimental and model simulation results is achieved, whereas a lower *R_k_* value is fitted compared to that in the unmodified Equation (9).

## 5. Conclusions

In the present work, the thermal diffusivity of a variety of polymer/carbonaceous nanocomposites, prepared with PLA and various types of LLDPE and reinforced with CNTs and a blend of GO/CNTs or GO/CNFs, has been experimentally studied. The zLLDPE/CNT nanocomposites demonstrate higher thermal diffusivity than the mLLDPE/CNT formulations. The results were in accord with the crystallinity content of the nanocomposites, thus revealing that the thermal diffusivity enhancement originates from two sources: the thermal diffusivity of the nanofillers and crystalline regions into the bulk. The zLLDPE/CNT thermal conductivity results were simulated by a nonlinear model resulting from Monte Carlo simulation and percolation theory. The fact that the results did not live up to expectations for much higher thermal diffusivity/conductivity may be attributed to the poor nanofiller dispersion, agglomerate formation and the high interfacial thermal resistance. Regarding the hybrid nanocomposites, enhanced thermal conductivity was obtained with increasing nanofiller loading, with the hybrid nanocomposites based on GO/CNTs exhibiting better performance than those based on GO/CNFs. Therefore, a synergistic effect between GO and CNTs has been assumed.

The thermal conductivity of the mLLDPE/GO/CNT and GO/CNF hybrids, as well as that of the PLA/GO/CNF formulations, was modeled by a linear model developed for hybrid nanocomposites, taken from the literature. In the modeling, an attempt was made to minimize the number of parameters, using the thermal conductivity values of the nanofillers from the literature and working in the range of the nominal dimensions of the CNTs, CNFs and GO nanoplatelets. Τhe important role of the interfacial resistance value in the model simulation was established.

The nonlinear trend of the thermal conductivity of the PLA/GO/CNT hybrids was simulated under the assumption of a 3D percolating network, which is formed by linking interactions between GO and CNTs, thereby supporting the heat flow.

Moreover, to incorporate the crystallinity increment with the nanofiller loading, a variable thermal conductivity value with varying nanofiller volume fractions was introduced into the employed thermal conductivity models.

In all cases it is emphasized in the text that, given the complex morphology of the materials under investigation, the experimental thermal conductivity results are governed by several factors, which either augment or impede thermal conductivity. Therefore, the experimental results represent a balance between the effects of these factors.

## Figures and Tables

**Table 1:** Crystallinity and thermal diffusivity of mLLDPE/CNT and zLLDPE/CNT nanocomposites.

Sample	Nanofiller Weight Fraction (%)	Crystallinity Degree (%)	Crystallinity Degree Increment (%)	Thermal Diffusivity (*α*, mm^2^/s) by LFA	Thermal Diffusivity Increment (%)
mLLDPE neat	-	18.7	-	0.130	-
mLLDPE + 2% CNT	2	19.0	1.6	0.143	10
mLLDPE + 4% CNT	4	20.4	9.1	0.160	23
mLLDPE + 6% CNT	6	18.6	-	0.169	30
mLLDPE + 8% CNT	8	20.0	7.0	0.180	38
mLLDPE + 10% CNT	10	20.1	7.5	0.188	44
zLLDPE neat	-	26	-	0.165	-
zLLDPE + 2% CNT	2	30.3	16.5	0.200	21
zLLDPE + 4% CNT	4	32.3	24.2	0.157	-
zLLDPE + 6% CNT	6	29.1	11.9	0.147	-
zLLDPE + 8% CNT	8	31.0	19.2	0.186	12
zLLDPE + 10% CNT	10	28.6	10.0	0.242	46
zLLDPE + 15% CNT	15	29.6	13.8	0.280	69
zLLDPE + 20% CNT	20	29.8	14.6	0.332	101

**Table 2:** Crystallinity and thermal diffusivity of mLLDPE/monofiller and hybrid nanocomposites.

Sample	Nanofiller Weight Fraction (%)	Crystallinity Degree (%)	Crystallinity Degree Increment (%)	Thermal Diffusivity (*α*, mm^2^/s) by LFA	Thermal Diffusivity Increment (%)
mLLDPE neat	-	24.6	-	0.13	-
mLLDPE + GO 1.31%	1.31	26.8	8.9	0.133	2.3
mLLDPE + CNT1.31%	1.31	24.8	0.81	0.148	13.8
mLLDPE + CNF 1.31%	1.31	26.3	6.9	0.132	1.5
mLLDPE + GO/CNF 1.31%	1.31	26.9	9.35	0.117	-
mLLDPE + GO/CNF 3.84%	3.84	27.3	11.0	0.137	5.4
mLLDPE + GO/CNF 6.25%	6.25	29.7	20.7	0.146	12.3
mLLDPE + GO/CNT 1.31%	1.31	24.6	-	0.142	9.2
mLLDPE + GO/CNT 3.84%	3.84	26.0	5.7	0.155	19.2
mLLDPE + GO/CNT 6.25%	6.25	25.6	4.0	0.157	20.7

**Table 3:** Crystallinity and thermal diffusivity of PLA/hybrid nanocomposites.

Sample	Nanofiller Weight Fraction (%)	Crystallinity Degree (%)	Crystallinity Degree Increment (%)	Thermal Diffusivity (*α*, mm^2^/s) by LFA	Thermal Diffusivity Increment (%)
PLA neat	-	4.4	-	0.112	-
PLA + GO/CNF 3.84%	3.84	3	-	0.112	-
PLA + GO/CNF 6.25%	6.25	4.2	-	0.127	13.4
PLA + GO/CNF 8.0%	8.0	9.8	122	0.136	21.4
PLA + GO/CNT 3.84%	3.84	18	309	0.141	25.9
PLA + GO/CNT 6.25%	6.25	12.6	186	0.151	34.8
PLA + GO/CNT 8.0%	8.0	10	127	0.155	38.4

**Table 4:** Experimental thermal conductivity results for the polymer nanocomposites under investigation.

Sample	Density *ρ* (g/cm^3^)	Heat Capacity *C_p_* J/g·K	Thermal Conductivity *K* (W/m·K) (δ*K* ± 0.003)
zLLDPE neat	0.918	1.51	0.228
zLLDPE + 2% CNT	0.924	1.40	0.250
zLLDPE + 4% CNT	0.925	1.60	0.233
zLLDPE + 6% CNT	0.930	1.63	0.223
zLLDPE + 8% CNT	0.940	1.50	0.261
zLLDPE + 10% CNT	0.940	1.18	0.269
zLLDPE + 15% CNT	0.970	1.33	0.362
zLLDPE + 20% CNT	0.982	1.29	0.420
mLLDPE neat	0.902	0.684	0.080
mLLDPE/GO/CNF 1.31%	0.990	0.684	0.079
mLLDPE/GO/CNF 3.84%	1.0	0.648	0.089
mLLDPE/GO/CNF 6.25%	0.970	0.645	0.091
mLLDPE/GO/CNT 1.31%	0.940	0.717	0.096
mLLDPE/GO/CNT 3.84%	0.980	0.693	0.105
mLLDPE/GO/CNT 6.25%	1.0	0.687	0.108
PLA neat	1.24	0.460	0.063
PLA/GO/CNF 3.84%	1.26	0.548	0.077
PLA/GO/CNF 6.25%	1.32	0.536	0.090
PLA/GO/CNF 8.0%	1.30	0.552	0.097
PLA/GO/CNT 3.84%	1.30	0.644	0.118
PLA/GO/CNT 6.25%	1.27	0.574	0.110
PLA/GO/CNT 8.0%	1.27	0.593	0.117

**Table 5:** Parameter values of the linear model (Equation (6)) for the thermal conductivity of the hybrid nanocomposites investigated.

Model Parameters	PLA/GO/CNF	mLLDPE/GO/CNF	mLLDPE/GO/CNT
*K_c_* (W/m∙K)	2000	2000	2000
*K_g_* (W/m∙K)	2000	2000	2000
*K_m_* (W/m∙K)	0.0631	0.08	0.08
*L_c_* (μm)	100	100	5.0
*L_g_* (μm)	5.0	5.0	5.0
*R_k_* (m^2^K/W)	2.0 × 10^−5^	2.0 × 10^−5^	1.5 × 10^−6^

**Table 6:** Nonlinear model (Equation (9)) parameters for the PLA/GO/CNT hybrid nanocomposites.

Model Parameters	PLA/GO/CNT
*K_c_* (W/m·K)	2000
*K_g_* (W/m·K)	2000
*L_c_* (μm)	5.0
*L_g_* (μm)	5.0
*R_k_* (m^2^K/W)	2.0 × 10^−5^
*β*	0.18

## Data Availability

The data that support the findings of this study are available from the corresponding author upon reasonable request.

## Supplementary Materials

The following supporting information can be downloaded at https://www.mdpi.com/article/10.3390/nano16181162/s1: Figure S1: SEM micrographs of mLLDPE/GO/CNF/1.31%. Reproduced with permission from reference [4]. Copyright Elsevier 2019. Figure S2: SEM micrographs of mLLDPE/GO/CNF/3.84%. Reproduced with permission from reference [4]. Copyright Elsevier 2019. Figure S3: SEM micrographs of mLLDPE/GO/CNF/6.25%. Reproduced with permission from reference [4]. Copyright Elsevier 2019. Figure S4: SEM micrographs of mLLDPE/GO/CNT/1.31%. R Reproduced with permission from reference [4]. Copyright Elsevier 2019. Figure S5: SEM micrographs of mLLDPE/GO/CNT/3.84%. Reproduced with permission from reference [4]. Copyright Elsevier 2019. Figure S6: SEM micrographs of mLLDPE/GO/CNT/6.25%. Reproduced with permission from reference [4]. Copyright Elsevier 2019. Figure S7: SEM micrographs of mLLDPE/GO/CNT/6.25%. Reproduced with permission from reference [4]. Copyright Elsevier 2019. Figure S8: Model simulation (Equation (6)) of thermal conductivity versus the nanofiller volume fraction for the mLLDPE/GO/CNT hybrid nanocomposites, for two sets of different values for *R_k_*, *L_c_* and *L_g_*. Figure S9: Model simulation (Equation (6)) of thermal conductivity versus nanofiller volume fraction for the mLLDPE/GO/CNT hybrid nanocomposites. The effect of the GO interfacial resistance *R_kg_* value on the nanocomposites’ thermal conductivity, keeping the CNT interfacial resistance *R_kc_* value constant. Figure S10: Model simulation (Equation (6)) of thermal conductivity versus nanofiller volume fraction for the mLLDPE/GO/CNT hybrid nanocomposites. The effect of the CNTs’ interfacial resistance *R_kc_* value on the nanocomposites’ thermal conductivity, keeping the GO interfacial resistance *R_kg_* value constant.
