# Supplementary material for: Experimental Study and Model Simulation of the Effective Thermal Conductivity of Polymer/Carbonaceous Nanocomposites
Source: Nanomaterials (Basel). 2026 Sep 15;16(18):1162. doi: 10.3390/nano16181162 (PMC13610368; doi:10.3390/nano16181162)
Supplement: Supplementary file 1 [file nanomaterials-16-01162-s001.zip › nanomaterials-4461112-supplementary.pdf]

Supplementary Material

for

Experimental Study and Model Simulation of the Effective Thermal Conductivity of Polymer/ Carbonaceous Nanocomposites

Panagiotis A. Klonos [1], Apostolos Kyritsis [1] and Evagelia Kontou [2,*]

[1] Physics Department, School of Applied Mathematical and Physical Sciences, National Technical University of Athens, 15780 Athens, Greece; pklonos@central.ntua.gr (P.A.K.); akyrits@central.ntua.gr (A.K.)
[2] Mechanics Department, School of Applied Mathematical and Physical Sciences, National Technical University of Athens, 15780 Athens, Greece
* Correspondence: ekontou@central.ntua.gr; Tel.: +30-2107721219

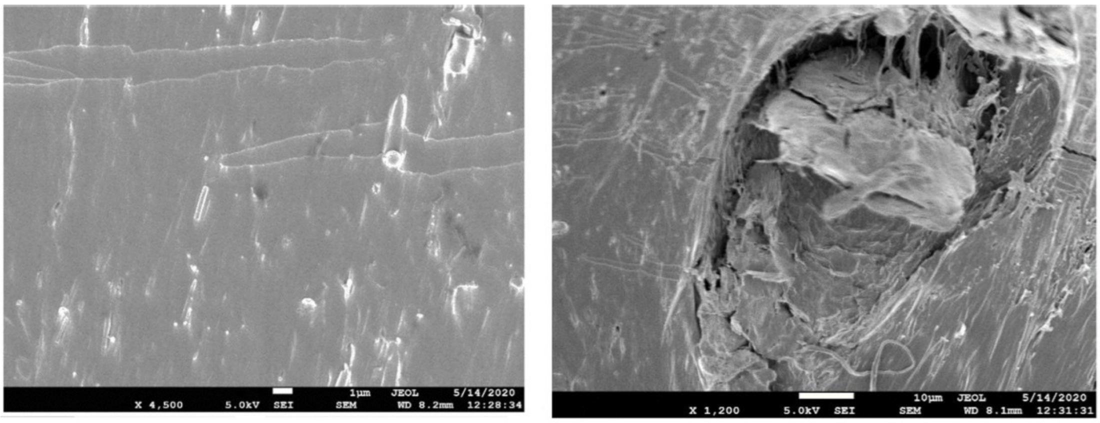

Figure S1. SEM micrographs for the mLLDPE/GO/CNF/1.31%. Reproduced with permission from reference [4]. Copyright Elsevier 2019.

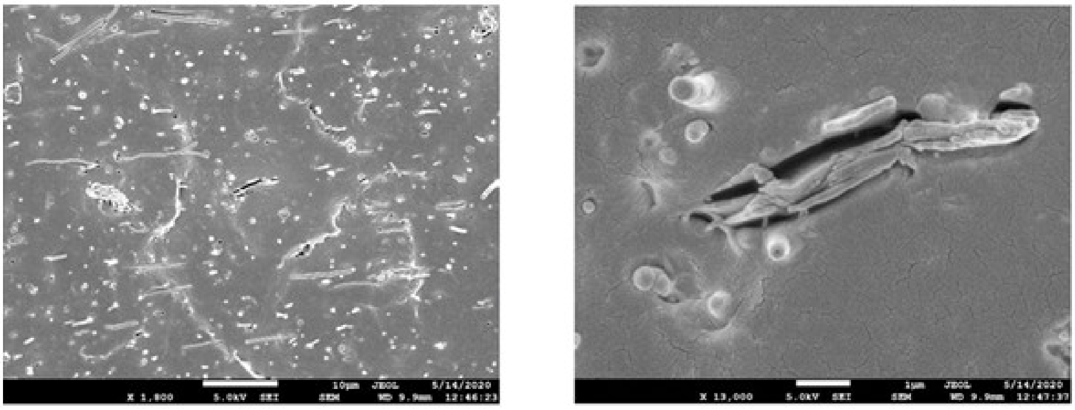

a) b)

Figure S2. SEM micrographs for the mLLDPE/GO/CNF/3.84%. Reproduced with permission from reference [4]. Copyright Elsevier 2019.

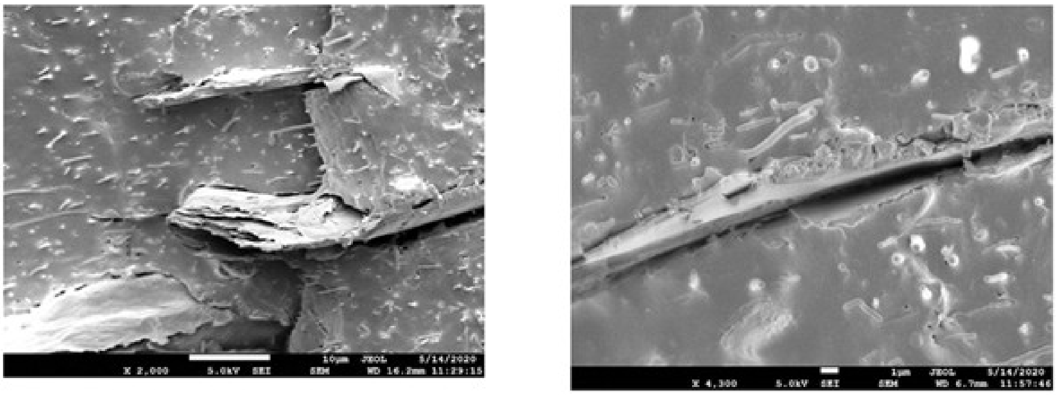

a) b)

Figure S3. SEM micrographs for the mLLDPE/GO/CNF/6.25%. Reproduced with permission from reference [4]. Copyright Elsevier 2019.

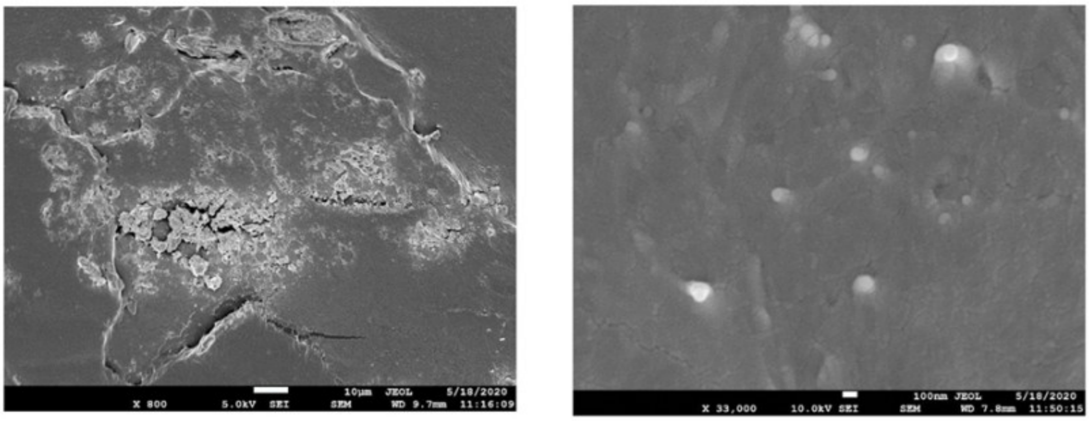

a) b)

Figure S4. SEM micrographs for the mLLDPE/GO/CNT/1.31%. R Reproduced with permission from reference [4]. Copyright Elsevier 2019.

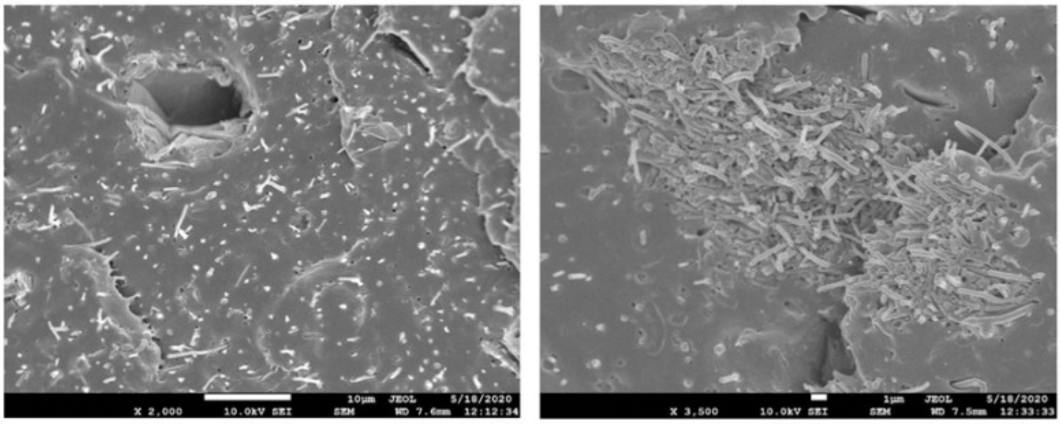

a) b)

Figure S5. SEM micrographs for the mLLDPE/GO/CNT/3.84%. Reproduced with permission from reference [4]. Copyright Elsevier 2019.

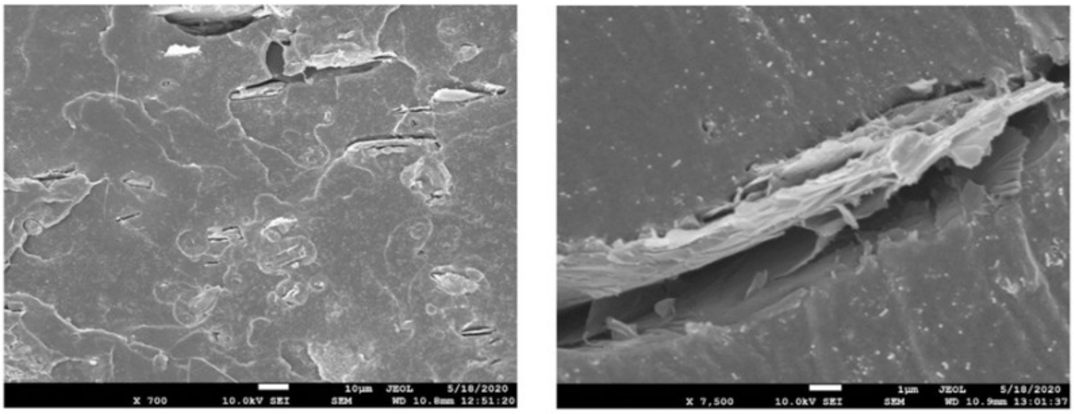

a) b)

Figure S6. SEM micrographs for the mLLDPE/GO/CNT/6.25%. Reproduced with permission from reference [4]. Copyright Elsevier 2019.

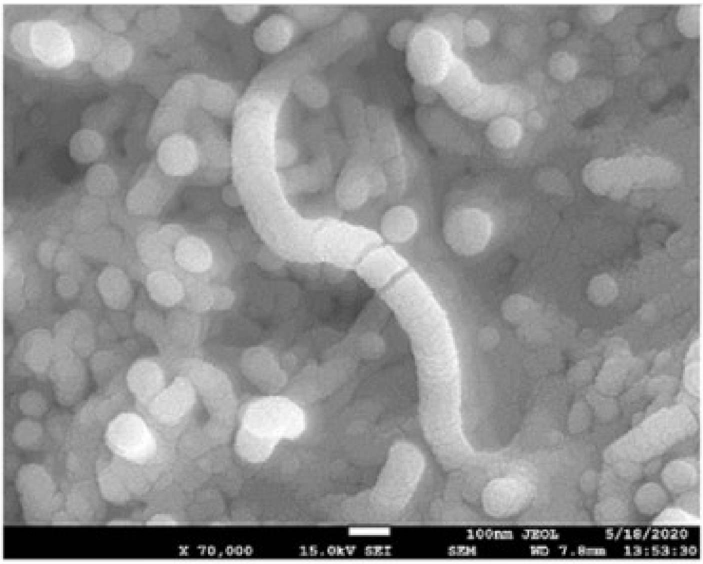

Figure S7. SEM micrographs for the mLLDPE/GO/CNT/6.25%. Reproduced with permission from reference [4]. Copyright Elsevier 2019.

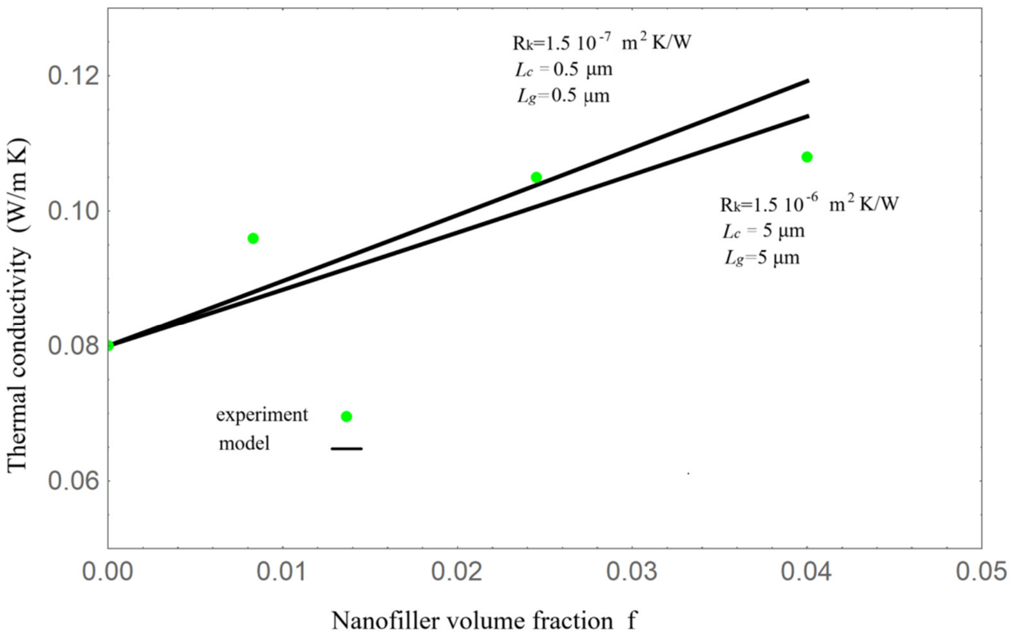

Figure S8. Model simulation (Eq. 6) of thermal conductivity versus nanofiller volume fraction for the mLLDPE/GO/CNT hybrid nanocomposites, for two sets of different values for R_k, L_c and L_g

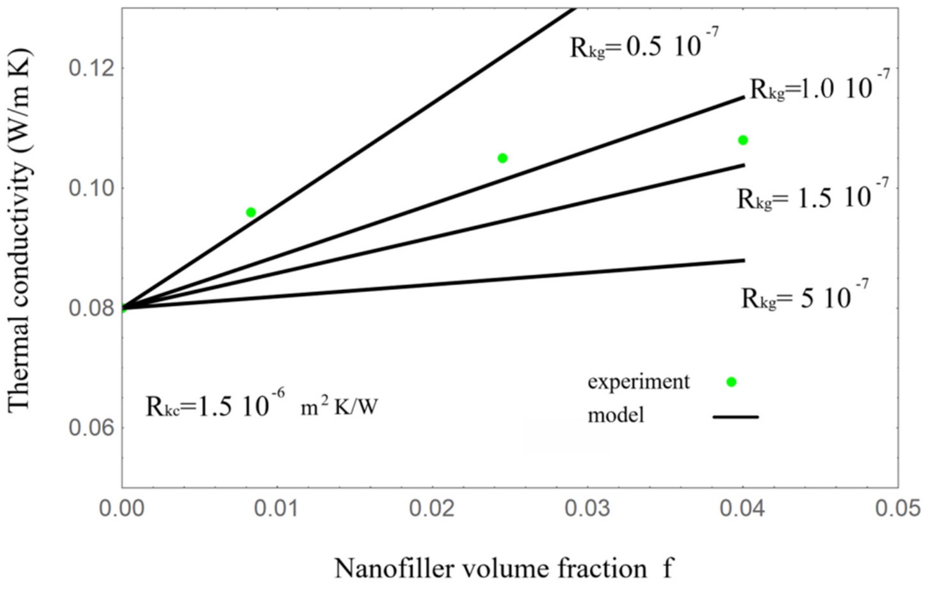

Figure S9. Model simulation (Eq. 6) of thermal conductivity versus nanofiller volume fraction for the mLLDPE/GO/CNT hybrid nanocomposites. The effect of the GO interfacial resistance R_{kg} value on the nanocomposites thermal conductivity, keeping the CNTs interfacial resistance R_{kc} value constant

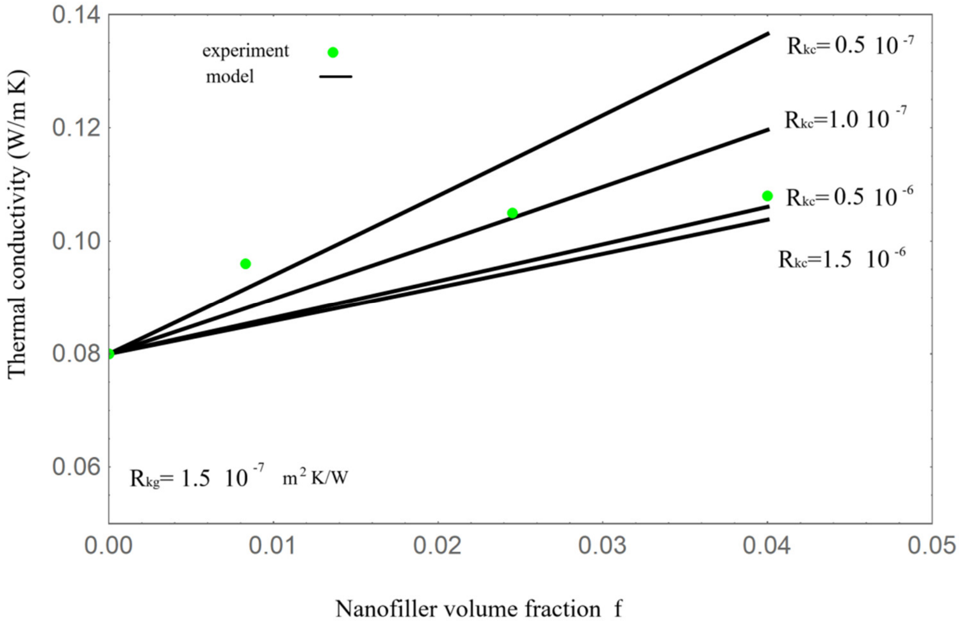

Figure S10. Model simulation (Eq.6) of thermal conductivity versus nanofiller volume fraction for the mLLDPE/GO/CNT hybrid nanocomposites. The effect of the CNTs interfacial resistance R_{kc} value on the nanocomposites' thermal conductivity, keeping the GO interfacial resistance R_{kg} value constant.
